# Development of a smartphone camera-based chemiluminescent lateral flow immunoassay for detecting African swine fever virus antibodies

**DOI:** 10.3389/fmicb.2025.1686382

**Published:** 2025-11-11

**Authors:** Yaning Sun, Suzhen Yang, Yunrui Xing, Lu Fan, Nannan Wang, Qingmei Li, Junqing Guo, Songlin Qiao, Gaiping Zhang

**Affiliations:** 1 Institute for Animal Health, Henan Academy of Agricultural Sciences, Zhengzhou, China; 2 Longhu Laboratory of Advanced Immunology, Zhengzhou, China

**Keywords:** African swine fever virus (ASFV) antibodies, chemiluminescent, lateral flow immunoassay, smartphone camera, on-site testing

## Abstract

African swine fever (ASF) is a highly contagious viral disease threatening global swine industries. Rapid and accurate detection of ASF virus (ASFV) antibodies is crucial for disease surveillance and control. The gold lateral flow immunoassay (GLFIA) is cost-effective and has been successfully applied in rapid on-site detection of ASFV. However, its sensitivity is relatively low. To enhance the detection sensitivity and accuracy while retaining convenience, we developed a chemiluminescent lateral flow immunoassay (CLFIA) for detecting ASFV antibodies based on the p72 trimer protein, which can immediately read the chemiluminescent signal through the camera of a smartphone. Compared with GLFIA and commercial enzyme-linked immunosorbent assay (ELISA), its sensitivity was improved by at least two orders of magnitude and nine orders of magnitude, respectively. Additionally, CLFIA shows no cross-reaction with antibodies from common swine disease viruses, and the detection results of 65 clinical samples have a 93.8% coincidence rate with those of commercial ELISA kits. This research successfully addressed the issue that traditional chemiluminescent detection relies on specialized instruments, providing a new technical approach for the highly sensitive and rapid detection of ASFV, and effectively promoting the development and application of CLFIA technology.

## 1 Introduction

African swine fever (ASF), caused by the African swine fever virus (ASFV), is an acute, febrile, and highly contagious disease affecting domestic and wild swine, with a very high case fatality rate (Bisimwa et al., 2024). Initially discovered in Kenya in 1909 (Gallardo et al., 2015), ASF has evolved from a localized African enzootic disease to a global pandemic. The current epizootic wave, dominated by genotype II strains (Giammarioli et al., 2024), first appeared in Georgia in 2007 (Oganesyan et al., 2013) and later spread to Eastern Europe in 2014 (Chenais et al., 2018), China in 2018 (Tao et al., 2020; Zhou et al., 2018), and Germany in 2020 (Desmecht et al., 2021). Recognized as a reportable disease by the World Organization for Animal Health (WOAH) (Hu et al., 2023), ASF has inflicted massive global economic damage (Dhollander et al., 2025), significantly destabilizing pork supply chains and threatening global protein availability. Because of the disease, China’s pig population has decreased by nearly 40% (Juszkiewicz et al., 2023; Wang et al., 2023).

Despite significant research effort (Dixon et al., 2019; Sunwoo et al., 2019), no safe and efficacious vaccine or antiviral therapy for ASF has been developed (Cui et al., 2024). To prevent the spread of the disease, early detection is crucial for the timely implementation of health and biosecurity control measures (Gallardo et al., 2015). Given the complex epidemiology and varied clinical manifestations of ASFV, rapid and reliable laboratory diagnostic methods are critically important. Animals infected with ASFV can survive for several weeks. However, some individuals who recover from acute infection may remain in a latent infection state, posing a risk of long-term viral shedding, while antibodies in their bodies can persist for a relatively long time (Mebus and Dardiri, 1980; Perez et al., 1998). Polymerase chain reaction (PCR) is the method of choice for the early detection of ASFV. It has demonstrated excellent sensitivity and specificity during the acute infection phase (Gallardo et al., 2015; Trinh et al., 2021). During the latent infection stage, serological antibody testing holds greater clinical significance. However, current on-site antibody testing products still have certain limitations, primarily in terms of sensitivity and potential cross-reactivity with related pathogens.

The key factors influencing the sensitivity and accuracy of ASF antibody tests are the selection and properties of the detection antigens, such as the structure, purity, and stability of recombinant proteins. The expression system is critical in determining antigen quality. Eukaryotic cell expression systems can perform post-translational modifications, such as protein folding, glycosylation, acylation, and phosphorylation, which render the expressed proteins more structurally similar to their natural counterparts, thereby enhancing their reactivity (Yang et al., 2025). p72 constitutes approximately 31%–33% of the total mass of ASFV virions (Liu et al., 2019) and is the most predominant structural component, existing in the viral capsid as a homotrimer (Zsak et al., 1993). It is also one of the first viral proteins linked to the induction of antibodies following infection. The preparation of the p72 trimer protein in its natural form is crucial for reducing false-positive reactions and improving the accuracy of antibody detection (Cubillos et al., 2013). Geng et al. (2022) developed an ASFV antibody test strip using colloidal gold-labeled p72 trimers produced by co-expressing p72/pB602L in human embryonic kidney 293 (HEK293) cells, demonstrating high sensitivity and accuracy for clinical and standard sera. Therefore, the preparation of p72 trimeric protein using eukaryotic expression systems has emerged as a pivotal strategy for improving the quality of ASFV antibody tests.

The new labeling materials can enhance the sensitivity of lateral flow immunoassays (LFIA). Previous studies have demonstrated the many advantages of chemiluminescent lateral flow immunoassay (CLFIA), such as low signal-to-noise ratio and enhanced sensitivity compared with gold lateral flow immunoassay (GLFIA) (Chen et al., 2016). Consequently, it has gathered significant research interest within the field of *in vitro* diagnostics (Deng et al., 2018; Jung et al., 2021). However, the CL signal cannot be directly observed with the naked eye, it requires the use of specialized or customized equipment for capture and analysis (Deng et al., 2018). Therefore, the development of a method that can conveniently analyze the results of CLFIA will significantly advance LFIA.

In this study, we developed a CLFIA using p72 trimers as capture antigens for ASFV antibody detection. Integrating smartphone-based image acquisition, this technology enables rapid, sensitive, and specific on-site testing of ASFV antibodies, providing an innovative solution that enhances both detection sensitivity and accuracy for point-of-care serological surveillance of ASFV.

## 2 Materials and methods

### 2.1 Materials

Lightning-Link HRP conjugation kit (ab102890) was purchased from Abcam Inc., (Cambridge, MA, USA). The enhanced chemiluminescence (ECL) substrate kit was purchased from NCM Biotec (Suzhou, China). Horseradish peroxidase (HRP)-conjugated antibody dilution buffer was purchased from Solarbio (Beijing, China). IgG-free/protease-free bovine serum albumin (BSA) was purchased from Jackson (West Grove, PA, USA). Recombinant staphylococcal protein A (r-SPA) was purchased from Nuptec (Hangzhou, China). The ASFV antibody ELISA test kit was purchased from JNT (Beijing, China).

Anti-DYKDDDDKG affinity resin was purchased from Gen Script (Nanjing, China). The HiLoad 16/600 Superdex 200 pg column was purchased from GE Healthcare (Uppsala, Sweden). Nitrocellulose (NC) membranes (HF13502S25, 30 × 2 cm^2^) and fiberglass were purchased from Millipore (Bedford, MA, USA). The purity of all other reagents used was of analytical grade or higher.

Positive sera for classical swine fever virus (CSFV), pseudorabies virus (PRV), and porcine circovirus type 2 (PCV-2) were procured from the China Institute of Veterinary Drug Control (Beijing, China). Positive sera with antibodies for ASFV, porcine epidemic diarrhea virus (PRRSV), porcine parvovirus (PPV) and foot-and-mouth disease virus (FMDV) were collected and stored at our laboratory. All serum samples were analyzed using the corresponding antibody detection kits provided by IDEXX, and the results confirmed the presence of specific antibodies. 65 field sera from various pig farms in Henan Province, China and were provided by our laboratory. Anti-p72 monoclonal antibody (p72 mAb), recombinant vectors pCMV-p72, and pCMV-B602L were prepared by our laboratory, as described previously (Geng et al., 2022). All sample treatments were strictly performed in accordance with the standard operating procedures for ASFV by OIE.

### 2.2 Expression, purification, and characterization of p72

The p72 trimer protein was prepared as described by Geng et al. (2022). The expression vectors pCMV-p72 and pCMV-B602L were co-transfected into HEK293 cells to facilitate protein expression. After 72 h, the cells were harvested by centrifugation and subsequently subjected to ultrasonic disruption. The resulting supernatant was purified via Flag affinity chromatography using anti-DYKDDDDKG affinity resin. The concentrated p72 protein was further separated using a HiLoad 16/600 Superdex 200 pg column and evaluated using sodium dodecyl sulfate polyacrylamide gel electrophoresis (SDS-PAGE). The protein concentration was determined using the bicinchoninic acid assay (BCA). ELISA was used to distinguish between ASFV antibody positive and negative sera and evaluate the antigenic activity of p72.

### 2.3 Preparation of the HRP-p72 probes

**FIGURE 1:**
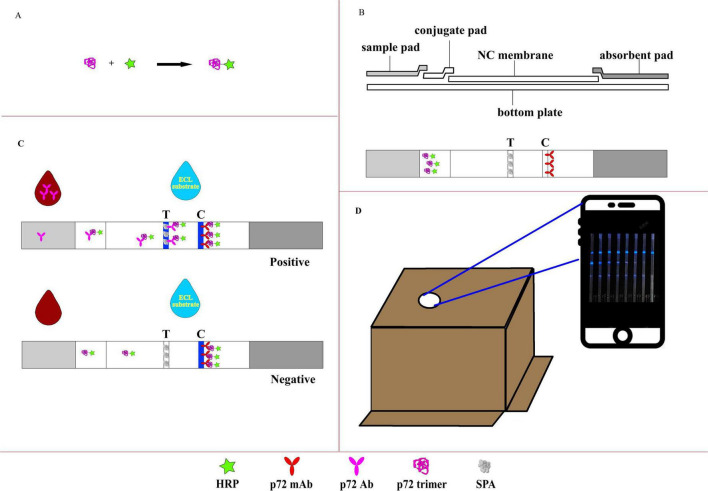
Illustration of the CLFIA. **(A)** Preparation of the CL probe; **(B)** basic structure of the CLFIA; **(C)** schematic of the CLFIA reaction principle; **(D)** result reading device.

The HRP-p72 probe was prepared following the manufacturer’s instructions for the Lightning-Link HRP kit (Figure 1A). Briefly, p72 trimer protein (100 μL; 0.965 mg/mL) was added to 10 μL modifier reagent and mixed gently. The p72 protein solution previously treated with the modifier reagent was aspirated using a pipette and directly added to the HRP mixture. The mixture was gently resuspended by aspirating and dispensing the liquid one or two times and then incubated in the dark at room temperature (20 °C–25 °C) for 3 h. After incubation, 10 μL quencher reagent was added to the p72 protein reaction tube and mixed gently. The resulting conjugated protein could be used after 30 min without further purification.

### 2.4 Preparation of CLFIA

The CLFIA is composed of NC membrane, conjugate pad, sample pad, absorbent pad, and PVC bottom plate (Figure 1B). To prepare the conjugate pad, an XYZ Dispensing platform (XYZ3050, BioDot, CA, USA) was used to evenly apply the HRP-p72 probe solution (80-fold dilution) at a rate of 7 μL/cm onto pre-treated glass fiber cotton (The buffer solution containing Na_2_B_4_O_7_⋅10H_2_O, BSA, PVP-10, and Triton X-100 was uniformly applied onto the glass fiber membrane and dried at 37 °C), which was then dried at 37 °C for 1 h. The same instrument was used to dispense SPA [0.75 mg/mL, phosphate-buffered saline (PBS)] and p72 monoclonal antibody (2 mg/mL, PBS) onto the NC membrane at a rate of 1 μL/cm, forming the test line (T line) and control line (C line), respectively, and dried at 42 °C for 2 h.

The NC membrane, conjugate pad, sample pad, absorbent pad, and bottom plate were assembled into a strip board. Then, the strip board was cut into 3.0 mm-wide test strips using a cutter (CM4000, BioDot, CA, USA) and stored in a sealed container away from light.

### 2.5 CLFIA testing procedure

The serum sample was diluted 1:200 with PBS, and 100 μL of the diluted solution was added to the reaction well. Insert the CLFIA into the sample solution and kept for 15 min. The ECL substrate (70 μL) was added to the center of the NC membrane. The bottom of the packaging box of the CLFIA, which has a pre-cut opening at the top for mobile phone camera capture, was opened, and the test strip was positioned beneath the box. The camera of the GT80 mobile phone (Honor, China) was aligned with the opening at the top of the box, and photos were taken within 5–13 min to determine the results.

The CLFIA reaction principle was showed in Figure 1C. When the sample flows through the conjugation pad, the anti-p72 antibody (Ab) in a positive sample binds with the p72-HRP probe to form an antigen antibody complex. Subsequently, this complex flows through the T line and is captured by the SPA. The unbound p72-HRP probe continues to migrate and is captured by the fixed P72 mAb at the C line. Then, the ECL substrate containing luminol is added, triggering the catalytic action of the HRP enzyme, producing visible blue light, which is captured by the camera in the darkness of the box (Figure 1D). A positive result is indicated when blue lines are observed at both the control (C) and the test (T) lines. A negative result is indicated when a clear blue line appears only at the C line, but none at the T line. Furthermore, color intensity at the T line indicates a positive correlation with the antibody titer within a certain range (Li et al., 2022).

## 3 Results and discussion

### 3.1 Expression, purification, and characterization of p72

**FIGURE 2:**
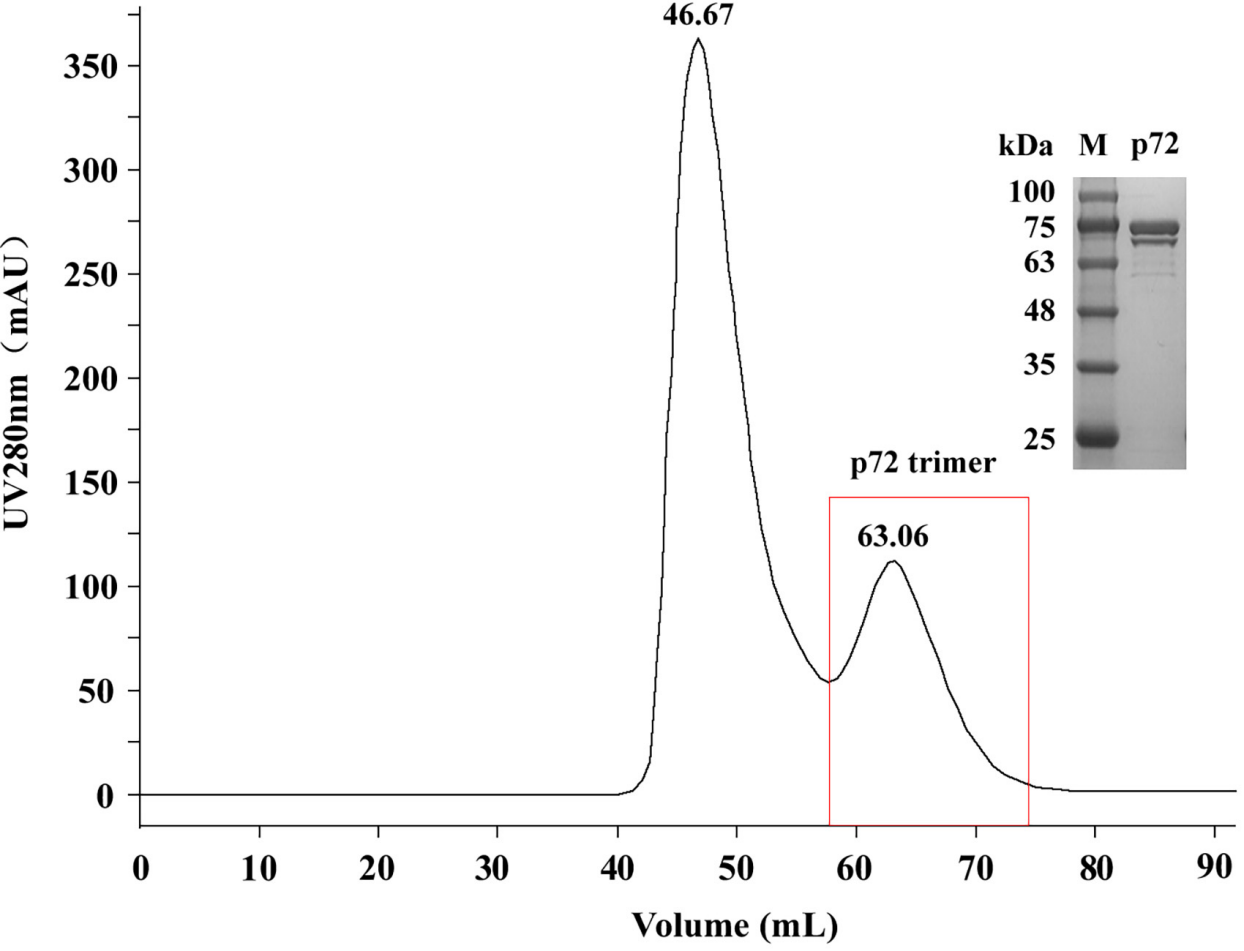
Sodium dodecyl sulfate polyacrylamide gel electrophoresis (SDS-PAGE) analysis of the p72 protein purified by gel filtration chromatography. M, protein molecular weight marker.

We prepared the p72 trimer by co-expressing the ASFV p72 protein and its chaperone protein pB602L in HEK293 cells. The product was purified by gel filtration chromatography. The elution peak position and molecular weight (approximately 70–100 kDa; Figure 2) were consistent with the results reported by Geng et al. (2022), thereby verifying the successful purification of the p72 trimer. The protein concentration obtained was 0.965 mg/mL.

Enzyme-linked immunosorbent assay plates were coated with different p72 trimer concentrations and then tested with ASFV negative and positive sera (Table 1). The optical density values at 450 nm (OD_450_) of the negative sera were all below 0.200, whereas those of positive sera were all above 1.000 under the three coating concentrations. Moreover, the OD_450_ values decreased as the coating concentration decreased, indicating the good antigenicity the prepared p72 trimer.

**TABLE 1:** Identification of p72 antigenicity by ELISA *n* = 3.

Sample no.	P72 trimer coating concentration (μg/mL)		
	2.4	1.2	0.6
54	0.129 ± 0.063	0.100 ± 0.061	0.067 ± 0.002
55	0.120 ± 0.064	0.114 ± 0.064	0.084 ± 0.004
56	0.098 ± 0.010	0.113 ± 0.065	0.102 ± 0.022
57	0.198 ± 0.037	0.190 ± 0.096	0.076 ± 0.008
68	0.152 ± 0.083	0.155 ± 0.139	0.095 ± 0.006
1	2.664 ± 0.106	1.746 ± 0.068	1.123 ± 0.171
2	3.292 ± 0.022	2.259 ± 0.087	1.429 ± 0.094
BC	0.042 ± 0.002	0.040 ± 0.004	0.031 ± 0.001

Samples 54, 55, 56, 57, and 68 were negative for African swine fever virus antibody, while samples 1 and 2 were positive. BC, blank control.

### 3.2 Selection of chemiluminescent probe mode and optimization of its amount

**FIGURE 3:**
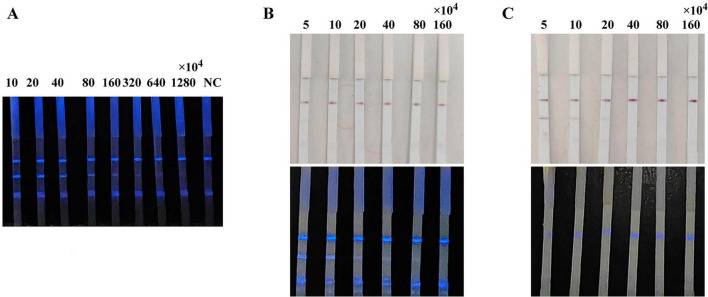
Comparison of the sensitivity of CLFIA using different probes. **(A)** CLFIA constructed based on the p72-HRP probe; **(B)** CLFIA constructed based on the Gold-p72-HRP probe; **(C)** CLFIA constructed based on the p72-Gold-HRP probe.

Chemiluminescent probes are key factors influencing the sensitivity of the CLFIA. To achieve higher sensitivity, this study designed three methods for preparing CL probes: directly coupling p72 with HRP to form a p72-HRP probe; colloidal gold labeling of the p72-HRP coupling to form a gold-p72-HRP probe; and simultaneously labeling the p72 and HRP monomers (molar ratio 1:1) with colloidal gold to form a p72-gold-HRP probe. The above probes were all added on the conjugation pad of the test strip, and the ASFV antibody-positive sera were detected. The results are shown in Figure 3. The CLFIA established with the p72-HRP probe did not produce a colloidal gold signal. The results could only be determined using a smartphone, with the detection sensitivity for positive serum reaching 320 × 10^4^. However, the results of CLFIA constructed based on the gold-p72-HRP and p72-gold-HRP probes could be judged by either naked-eye observation of the colloidal gold signal or with a smartphone. The colloidal gold detection sensitivity of the gold-p72-HRP probe mode is 5 × 10^4^, while the CL detection sensitivity was 20 × 10^4^. Furthermore, the colloidal gold detection sensitivity of the p72-gold-HRP probe mode was 20 × 10^4^, while the CL detection sensitivity was less than 5 × 10^4^. Given the widespread adoption of smartphones, the convenience of smartphone camera for result detection is comparable to the visual detection of colloidal gold signals. Therefore, sensitivity becomes an important criterion for evaluating probes; the p72-HRP probe is preferred, as its sensitivity is at least four orders of magnitude higher than that of the other two probes.

**FIGURE 4:**
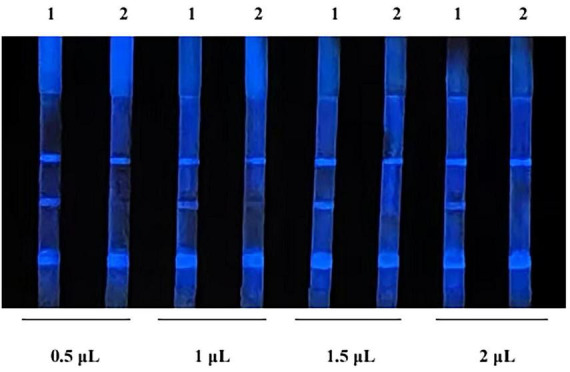
Optimization of probe amount. 1, indicates negative samples; 2, indicates positive samples.

The results of the test using different amount of CL probe are shown in Figure 4. When the probe was 0.5 μL per strip (20-fold dilution), the background color was the lightest, showing optimal intensity and duration. Therefore, it is determined that the optimal amount of probe is 0.5 μL per strip.

### 3.3 Determination of the shooting time

**FIGURE 5:**
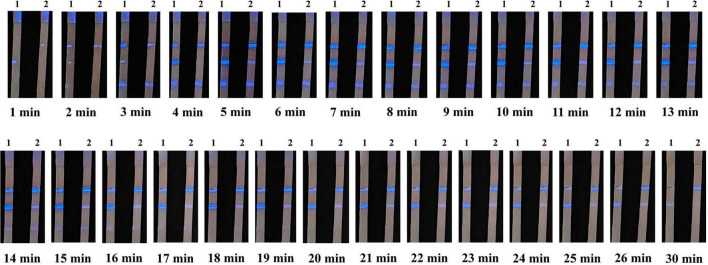
Images of CLFIA results captured at different times. Strips 1 and 2 represent the ASFV antibody-positive serum at the 1 × 10^4^ and 20 × 10^4^ dilutions.

Chemiluminescent lateral flow immunoassay detected ASFV antibody-positive sera. Images were captured at 1 min intervals after the addition of the ECL substrate (Figure 5). Color could be judged after 5 min, and the signal remained stable between 5 and 13 min. However, the signal began to weaken after 14 min and almost completely disappeared at 30 min. Therefore, the reading of results should be performed within 5–13 min after adding the ECL substrate to ensure the accuracy and repeatability of the CLFIA.

### 3.4 CLFIA sensitivity testing

**FIGURE 6:**
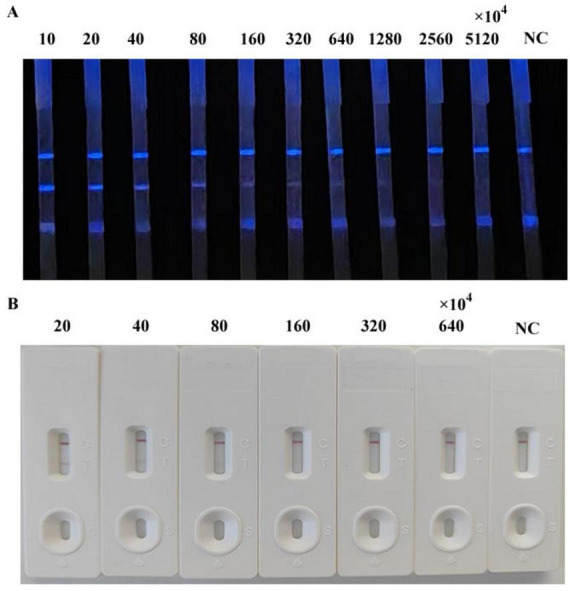
Titers of ASFV antibody-positive sera. **(A)** CLFIA. **(B)** GLFIA.

We simultaneously evaluated the detection sensitivity of the CLFIA, GLFIA and the commercial ASFV antibody ELISA test kit using ASFV antibody-positive serum titers (Figure 6). The CLFIA titer for the ASFV antibody-positive serum was 1:320 × 10^4^, the GLFIA titer was 1:80 × 10^4^, and the ELISA titer was 1:6400. CLFIA exhibited the highest sensitivity, which was approximately two orders of magnitude higher than that of GLFIA and nine orders of magnitude higher than that of ELISA.

### 3.5 The specificity of CLFIA

**FIGURE 7:**
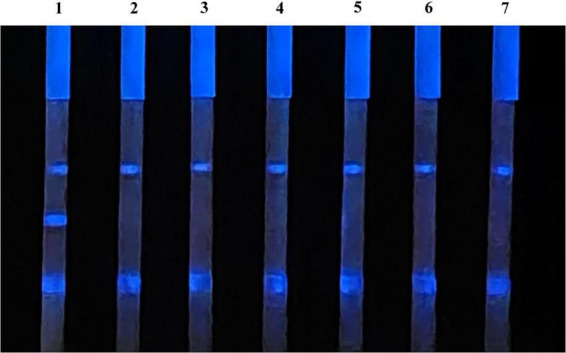
1, ASFV antibodies-positive serum; 2, PRRSV antibodies-positive serum; 3, PPV antibodies-positive serum; 4, PCV-2 antibodies-positive serum; 5, PRV antibodies-positive serum; 6, CSFV antibodies-positive serum; 7, ASFV antibodies-negative serum.

Chemiluminescent lateral flow immunoassay was used to detect the positive sera with antibodies of ASFV, PRRSV, PPV, PCV-2, PRV, and CSFV, as well as the ASFV antibodies negative sera, to evaluate its specificity (Figure 7). The CLFIA only showed a positive result for ASFV antibody-positive sera, with a negative result for all the other sera. Therefore, the ASFV antibody CLFIA developed in this study demonstrated good specificity.

### 3.6 Clinical sample detection

Furthermore, 65 clinical pig serum samples were tested using CLFIA and commercial ASFV antibody ELISA test kit, to evaluate the feasibility of CLFIA in clinical diagnosis (Table 2). All 21 positive samples detected by ELISA kits were also positive in CLFIA, with a positive coincidence rate of 100%. Furthermore, the ELISA kit detected 44 negative samples, while the CLFIA detected 40 negative samples, with a negative coincidence rate of 90.9%. The overall coincidence rate of the two methods was 93.8%. The two assays differed in results for 4 samples, which were negative on ELISA but positive on CLFIA. These 4 samples were collected from two different pig farms, both of which returned other positive samples, indicating they are likely ASFV-positive pig farms. Given that the detection sensitivity of the CLFIA is significantly higher than that of the ELISA, its positive detection rate is theoretically expected to be correspondingly higher. Furthermore, considering that these four serum samples originated from positive pig farm, the results indicate that the CLFIA detection method developed in this study has high accuracy and is thus suitable for the rapid screening of clinical samples.

**TABLE 2:** Comparison between ELISA and CLFIA for detecting ASFV.

	ELISA kits		
CLFIA	Negative	Positive	Total
Negative	40	0	40
Positive	4	21	25
Total	44	21	65

## 4 Discussion

African swine fever virus infection has multiple manifestations, including the peracute, acute, subacute, chronic, and asymptomatic forms. Among them, the acute form is the most common, with a fatality rate as high as 100%, posing a serious threat to the global swine industry. The incubation period in natural infections of ASF is typically 4–19 days. Antibodies can be detected approximately 7–9 days after infection and can be detected for the rest of the animal’s life. For the peracute, acute and subacute forms of ASF infection, nucleic acid testing is the preferred detection method (Gallardo et al., 2021). Antibody detection plays a crucial role in subacute or chronic infections, in the recovery of infected animals, and in the thorough elimination of ASF from pig farms (Li et al., 2020). Currently, the commonly used antibody detection methods include ELISA (Yu et al., 2021) and LFIA (Hu et al., 2023), which are suitable for different scenarios. The ELISA method requires professional technicians and laboratory equipment and is suitable for high-throughput screening in large-scale pig farms (Yang et al., 2022); while the LFIA method is simple to operate, does not require specialized equipment or personnel, and is more suitable for on-site rapid detection in medium and small-sized pig farms. Colloidal gold has traditionally served as the labeling material in LFIA due to its favorable stability, ability to provide visualized results, and widespread applicability.

However, its relatively low detection sensitivity has increasingly limited its ability to meet assay requirements. Multiple studies have shown that GLFIA methods utilizing the p72 protein achieve analytical sensitivities within the range of 1:64–1:10,000 (Aira et al., 2023; Geng et al., 2022; Wan et al., 2022; Zhu et al., 2022). The LFIA for ASFV antibodies established with quantum dots (Niu et al., 2022), fluorescent microspheres (Li et al., 2022), and CL (Yang et al., 2021) can improve the detection performance of the LFIA to a certain extent. However, reliance on instrument equipment for result interpretation may weaken the advantage of operational simplicity in the LFIA to some degree. Miao et al. (2024) designed two chemiluminescence immunoassay (CLIA) methods for identifying antibodies against the ASFV p72 antigen. These included a conventional plate-based blocking CLIA (p72-CLIA) and a magnetic particle-based tubular competitive CLIA (p72-MPCLIA). The p72-MPCLIA approach significantly decreased the assay duration to 15 min and supported complete automation of the detection process. The application scenarios of CLIA and CLFIA are different. CLIA relies on automated instrumentation to enable high-throughput screening of large sample batches, making it suitable for laboratory settings. In contrast, CLFIA does not require specialized detection instruments, which renders it more appropriate for visual, on-site, and real-time detection in resource-limited or field environments.

## 5 Conclusion

In this study, we developed a p72 protein-based CLFIA to detect ASFV antibodies in serum using a smartphone. The sensitivity of this method was two orders of magnitude higher than that of GLFIA and nine orders of magnitude higher than that of a commercial ELISA test kit. It exhibited no cross-reaction with antibodies of common swine diseases and has a detection coincidence rate of up to 93.8% with a commercial ELISA test kit. Therefore, this technology demonstrates potential applications in the clinical detection of ASFV. With our CLFIA, the CL signal could be read in real time using a smartphone camera, effectively eliminating the need for technical CL instruments. Our study demonstrates the utility of the CLFIA and highlights its potential for on-site real-time detection for clinical diagnosis, food safety, and other applications.

## Data Availability

The original contributions presented in this study are included in this article/supplementary material, further inquiries can be directed to the corresponding author.
